# Emergence and Persistent Dominance of SARS-CoV-2 Omicron BA.2.3.7 Variant, Taiwan

**DOI:** 10.3201/eid2904.221497

**Published:** 2023-04

**Authors:** Pei-Lan Shao, Hsiao-Chen Tu, Yu-Nong Gong, Hung-Yu Shu, Ralph Kirby, Li-Yun Hsu, Hui-Yee Yeo, Han-Yueh Kuo, Yi-Chia Huang, Yung-Feng Lin, Hui-Ying Weng, Yueh-Lin Wu, Chien-Chih Chen, Tzen-Wen Chen, Kuo-Ming Lee, Chung-Guei Huang, Shin-Ru Shih, Wei J. Chen, Chen-Chi Wu, Chong-Jen Yu, Shih-Feng Tsai

**Affiliations:** National Taiwan University Hospital Hsin-Chu Branch, Hsin-Chu, Taiwan (P.-L. Shao, L.-Y. Hsu, H.-Y. Yeo, H.-Y. Kuo, Y.-C. Huang, C.-C. Wu, C.-J. Yu);; Institute of Molecular and Genomic Medicine, National Health Research Institutes, Miaoli, Taiwan (H.-C. Tu, Y.-F. Lin, S.-F. Tsai);; Research Center for Emerging Viral Infections, Chang Gung University College of Medicine, Taoyuan, Taiwan (Y.-N. Gong, K.-M. Lee, C.-G. Huang, S.-R. Shih);; Linkou Chang Gung Memorial Hospital, Taoyuan (Y.-N. Gong, K.-M. Lee, C.-G. Huang S.-R. Shih);; Chang Gung University College of Medicine, Taoyuan (Y.-N. Gong, K.-M. Lee, C.-G. Huang, S.-R. Shih);; Chang Jung Christian University, Tainan, Taiwan (H.-Y. Shu);; National Yang Ming Chiao Tung University, Taipei, Taiwan (R. Kirby, H.-Y. Weng, S.-F. Tsai);; National Yang-Ming University, Taipei (H.-Y. Weng);; TMU Research Center of Urology and Kidney, Taipei Medical University, Taipei, Taiwan (Y.-L. Wu);; Wei-Gong Memorial Hospital, Miaoli, Taiwan (Y.-L. Wu, C.-C. Chen, T.-W. Chen,);; Center for Neuropsychiatric Research, National Health Research Institutes, Miaoli (W.-J. Chen);; Institute of Epidemiology and Preventive Medicine, National Taiwan University College of Public Health, Taipei (W.-J. Chen);; National Taiwan University College of Medicine, Taipei (C.-C. Wu)

**Keywords:** COVID-19, Omicron BA.2.3.7, Taiwan, coronavirus disease, SARS-CoV-2, severe acute respiratory syndrome coronavirus 2, viruses, respiratory infections, zoonoses, vaccine-preventable diseases

## Abstract

Since April 2022, waves of SARS-CoV-2 Omicron variant cases have surfaced in Taiwan and spread throughout the island. Using high-throughput sequencing of the SARS-CoV-2 genome, we analyzed 2,405 PCR-positive swab samples from 2,339 persons and identified the Omicron BA.2.3.7 variant as a major lineage within recent community outbreaks in Taiwan.

The COVID-19 pandemic, caused by SARS-CoV-2, originated in China in late 2019, probably in the city of Wuhan (*1*,*2*). The outbreak of this unusual respiratory disease led to a wide variety of responses by various countries across the world (*3*–*6*). The response in Taiwan was rapid and based on both its proximity to China and its experiences during the SARS pandemic ≈2 decades earlier (*5*,*7*,*8*). The introduction of strict travel restrictions on incoming air and sea passengers, long compulsory quarantine periods for the few residents allowed to enter Taiwan, and a vast public acceptance of safety measures (e.g., social distancing, temperature checks, mask wearing) resulted in a delay in the emergence of the COVID-19 pandemic in Taiwan compared with other countries (*5*,*9*,*10*). Until April 2022, there were only limited outbreaks, all of which were quickly contained. Taiwan therefore provides a unique opportunity to explore what happened when the Omicron variant finally evaded the controls put in place by the Taiwan government and began to spread through the country’s population. 

Residents of Taiwan had not been exposed, on a large scale, to any of the virus variants before Omicron. By the time SARS-CoV-2 began to spread widely in Taiwan April 2022, there had been around 17,000 recorded cases of COVID-19 in the country, and most of them were linked to the Alpha variant (almost all cases in our study had not been infected with SARS-CoV-2 before). Vaccination rates of Taiwan’s population at that time were 82.7% having received 1 dose, 78% having received 2 doses, and 59.1% having received 3 doses. The vaccines used in Taiwan before May 2022 were the Oxford-AstraZeneca vaccine (https://www.astrazeneca.com), the Pfizer-BioNTech vaccine (https://www.pfizer.com), the Moderna vaccine (https://www.modernatx.com), the Johnson & Johnson/Janssen vaccine (https://www.jandj.com), and The Median vaccine (a protein subunit COVID-19 vaccine made in Taiwan). Most residents of Taiwan received doses of the first 3 vaccines.

Very few COVID-19 cases occurred in Taiwan during 2020 and 2021. Clustered infections were reported in May and June 2021, mainly in northern Taiwan. Even at the peak, only hundreds of positive cases were recorded by Taiwan’s Centers for Disease Control. Early in 2022, several Omicron infection clusters were noted, first in northern Taiwan, and new cases quickly followed, soon exceeding 50,000 per day, with outbreaks affecting the entire country (Figure 1) (Infectious Disease Statistics Query System, https://nidss.cdc.gov.tw/nndss/disease?id=19CoV). 

**Figure 1:**
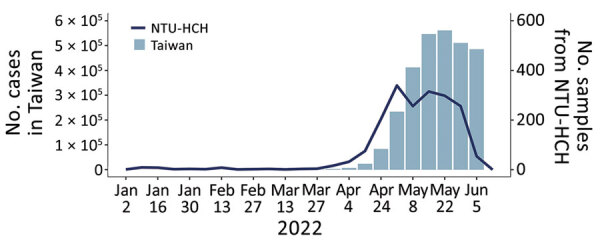
Weekly statistics for confirmed COVID-19 cases in Taiwan and sequenced samples, lineage distribution, and mutation prevalence derived from the NTU-HCH surveillance program, January–June 2022. Graph shows the number of COVID-19 confirmed cases in Taiwan and the sequenced samples from NTU-HCH from January (epidemiologic week 1) to early June (epidemiologic week 23). This figure was constructed using the publicly available data of Taiwan Centers for Disease Control (https://nidss.cdc.gov.tw/nndss/disease?id=19CoV). NTU-HCH, National Taiwan University Hospital–Hsinchu Branch.

## The Study

**Figure 2:**
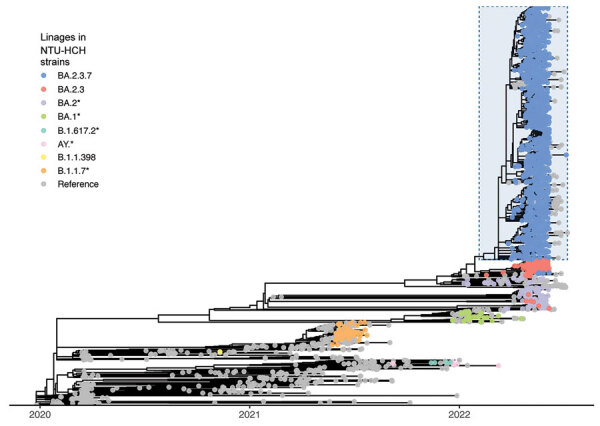
Phylogenetic analysis of SARS-CoV-2 sequences based on 1,966 sequences from the NTU-HCH surveillance program in Taiwan and 881 sequences from GISAID (https://www.gisaid.org). Lineages of NTU-HCH strains are annotated in different colors; asterisks (*) represent the collection of a specific lineage with its sublineage. The BA.2.3.7 strains were dominantly circulating in Taiwan from March 2022, highlighted by light blue in the tree. NTU-HCH, National Taiwan University Hospital–Hsinchu Branch.

To gain insights into community transmission and to monitor viral evolution, we deployed a genomic surveillance protocol at National Taiwan University Hospital Hsinchu Branch (NTU-HCH) whereby we performed whole-genome sequencing on nasal swab samples detected by PCR to be positive for SARS-CoV-2 (Appendix 1). To ensure data quality, we submitted genomic data to GISAID (https://www.gisaid.org) only on those sequences that had >98% coverage of the 29,903-bp SARS-CoV-2 target genome. We used the same set of high-quality sequences for tracking the signature mutations in the viral samples (Table 1) and for phylogenetic analysis (Figure 2; Appendix 1, Figure 1). We found 2,405 samples among 5 batches that met the above criterion and this generated 2,043 sequences (84.9% pass rate). We selected 1,966 sequences for GISAID submission (Appendix 1 Table 1).

**Table 1:** Signature mutations in SARS-CoV-2 BA.2.3.7 sublineages of viral samples from a study of the Omicron BA.2.3.7 variant in community outbreaks, Taiwan

Lineage	Accumulated mutations*			Selected mutations†
	BA.2	BA.2.3	BA.2.3.7	
ORF1a	S135R	S135R	S135R	NA
	NA	NA	NA	A591V
	NA	NA	L631F	L631F
	T842I	T842I	T842I	NA
	NA	NA	NA	I1091T
	G1307S	G1307S	G1307S	NA
	NA	A2909V	A2909V	NA
	L3027F	L3027F	L3027F	NA
	T3090I	T3090I	T3090I	NA
	L3201F	L3201F	L3201F	NA
	NA	NA	NA	T3224A
	T3255I	T3255I	T3255I	NA
	P3395H	P3395H	P3395H	NA
	del3675	del3675	del3675	NA
	del3676	del3676	del3676	NA
	del3677	del3677	del3677	NA
	NA	NA	NA	V3683I
Spike	T19I	T19I	T19I	NA
	L24S	L24S	L24S	NA
	del25	del25	del25	NA
	del26	del26	del26	NA
	del27	del27	del27	NA
	NA	NA	K97E	K97E
	G142D	G142D	G142D	NA
	V213G	V213G	V213G	NA
	G339D	G339D	G339D	NA
	S371F	S371F	S371F	NA
	S373P	S373P	S373P	NA
	S375F	S375F	S375F	NA
	T376A	T376A	T376A	NA
	D405N	D405N	D405N	NA
	R408S	R408S	R408S	NA
	K417N	K417N	K417N	NA
	N440K	N440K	N440K	NA
	S477N	S477N	S477N	NA
	T478K	T478K	T478K	NA
	E484A	E484A	E484A	NA
	Q493R	Q493R	Q493R	NA
	Q498R	Q498R	Q498R	NA
	N501Y	N501Y	N501Y	NA
	Y505H	Y505H	Y505H	NA
	D614G	D614G	D614G	NA
	H655Y	H655Y	H655Y	NA
	N679K	N679K	N679K	NA
	P681H	P681H	P681H	NA
	N764K	N764K	N764K	NA
	D796Y	D796Y	D796Y	NA
	Q954H	Q954H	Q954H	NA
	N969K	N969K	N969K	NA
	NA	NA	NA	G1251V
ORF3a	NA	L140F	L140F	NA
	T223I	T223I	T223I	NA
Nucleocapsid	P13L	P13L	P13L	NA
	del31	del31	del31	NA
	del32	del32	del32	NA
	del33	del33	del33	NA
	R203K	R203K	R203K	NA
	G204R	G204R	G204R	NA
	NA	NA	M322I	M322I
	S413R	S413R	S413R	NA

*Using SARS-CoV-2 Wuhan-Hu-1 (GenBank accession no. MN908947.3) as the reference sequence. Mutations in ORF1b, E, M, ORF6, and ORF8 are not shown as they are identical between BA.2, BA.2.3 and BA.2.3.7. NA, not applicable; ORF, open reading frame. †Mutations detected in the Omicron lineages are characterized in this study.

We analyzed the assembled viral genome sequences (Appendix 1 Table 2) and tracked the lineages and nonsynonymous amino acid changes in the Omicron samples collected during 2022 (Appendix 1 Figures 2, 3). Comparing the later 3 datasets (batches 3–5), we discovered that 3 amino acid changes (open reading frame [ORF]1a: L631F; spike (S): K97E; nucleocapsid; M322I) occurred only after the fourth sequencing batch. The percentage of sequences containing the signatures progressed steadily from 62% in batch 4 to 85% in batch 5. All batch-3 isolates belonged to the BA.1 or BA.2 classification, suggesting that the rapid increase of cases in Taiwan in April and May 2022—from 0 cases/day to ≈100,000 cases/day—came from a strain (BA.2.3.7) that might have been involved in a founder effect.

To construct the framework of the phylogenetic tree, we took 1,966 genome sequences from our study and analyzed them in the global context of 881 GISAID reference sequences (Figure 2; Appendix 2). We then zoomed in and compared the 1,577 Omicron sequences of our study against the 228 Omicron BA.2.3.7 strains from GISAID. Those sequences were reported from 21 countries, including 51 from Taiwan (Table 2). We conducted phylogenetic analysis using the Pango-dynamic nomenclature system (*11*).

**Table 2:** Submitted sequences of SARS-CoV-2 BA2.3.7 from different countries for inclusion in a study of the Omicron BA.2.3.7 variant in community outbreaks, Taiwan

Country	No. sequences
Taiwan	51*
Japan	44
United States	37
Indonesia	27
Hong Kong	22
Australia	7
Denmark	6
Canada	5
Singapore	4
South Korea	4
Philippines	3
Thailand	3
France	3
Cambodia	2
Austria	2
Sweden	2
Germany	2
Spain	1
Vietnam	1
Slovenia	1
New Zealand	1
Total	228

*Number does not include the contributions from this study.

We found evidence that this novel lineage BA.2.3.7 with 3 amino acid changes (ORF1a: L631F; S: K97E; and nucleocapsid: M322I) was circulating dominantly in Taiwan over the study period. Of note, the first BA.2.3.7 strain identified in the epidemic in Taiwan was collected on March 27, 2022, and since that time we detected several genomic changes affecting this Omicron lineage. For example, we noted a new mutation, G1251V (Appendix 1 Figure 3, green line) in the S protein, from April onward, and that particular circulating lineage then rapidly spread across Taiwan.

## Conclusions

We acknowledge that our study is limited in that we conducted the genomic surveillance in only 1 medical center; therefore, the observed dominance of BA.2.3.7 might be due to clustering of cases. Of note, while this paper was in preparation, we became aware that several viral sequences with the same signature mutations had been reported in Taiwan. Although the number of cases was relatively small (51) compared with the number of cases we studied, the 4 locations in Taiwan reporting those cases were different from our collection point at the hospital. Thus, this new lineage appeared to be broadly detectable across Taiwan. 

Other Asia-Pacific countries have also recently reported a substantial cumulative prevalence of the BA.2.3.7 variant (Table 2). Among the 44 Omicron BA.2.3.7 strains reported from Japan, 2 of the affected persons had travel history to Vietnam and 41 to Taiwan, suggesting considerable silent outward transmission from Taiwan. In contrast, BA.2.3.7 accounts for <0.5% of the sequences reported in either California, USA, or globally. The emergence of Omicron BA.2.3.7 in Asia is remarkable. Because there are no reliable genomic data from early cases in Malaysia and Vietnam, our phylogenic analysis and the metadata from GISAID suggests that travel between countries in Asia contributed to the rapid spread of this unique Omicron lineage. 

In summary, our genomic dataset is uniquely valuable for understanding how a major COVID-19 outbreak occurs in a naive and vaccinated population in Taiwan, a country with a very limited number of entry events. We theorize that the dominant circulation of BA.2.3.7 in Taiwan is likely the result of genetic drift or a founder effect, although it is also possible that increased transmissibility or vaccine evasion played some part. As countries in Asia move from zero tolerance to more open COVID-19 policies, continued surveillance of SARS-CoV-2 using next-generation sequencing is important. Early detection of viral evolution events in endemic areas will help minimize future disruptions caused by a new variant.

Appendix 1 Additional materials and methods for emergence and persistent dominance of SARS-CoV-2 Omicron BA.2.3.7 variant, Taiwan.

Appendix 2 Sequences retrieved from GISAID and GenBank for emergence and persistent dominance of SARS-CoV-2 Omicron BA.2.3.7 variant, Taiwan.
