# Supplementary material for: Emergence and Persistent Dominance of SARS-CoV-2 Omicron BA.2.3.7 Variant, Taiwan
Source: Emerg Infect Dis. 2023 Apr;29(4):792–6. doi: 10.3201/eid2904.221497 (PMC10045703; doi:10.3201/eid2904.221497)
Supplement: Appendix 1 — Additional materials and methods for emergence and persistent dominance of SARS-CoV-2 Omicron BA.2.3.7 variant, Taiwan. [file 22-1497-Techapp-s1.pdf]

Article DOI: http://doi.org/10.3201/eid2904.221497

EID cannot ensure accessibility for supplementary materials supplied by authors. Readers who have difficulty accessing supplementary content should contact the authors for assistance.

Emergence and Persistent Dominance of Omicron BA.2.3.7 Variant, Taiwan

Appendix 1

Additional Material and Methods

METHODS

We applied Illumina COVIDSeq amplicon sequencing technology to the whole-genome analysis of SARS-CoV-2. RNA was extracted from virus-inactivated swab samples. Complementary DNA synthesis was carried out using the reagents provided by the manufacturer, by which the SARS-CoV-2 genome underwent reverse transcription and was then amplified in 98 overlapping amplicons, together with appropriate human controls. In the final optimized procedure, we processed 384 samples at a time, and analyzed the pooled libraries on a single lane of a S4 chip using a NovaSeq6000.

Patients and RNA extraction

Representative samples were collected from National Taiwan University Hospital Hsinchu Branch (NTU-HCH). Before the major outbreak, nearly 300 samples were surveyed in January-April, 2022. On the other hand, we sequenced close to 2000 samples during the major outbreak in May, representing approximately 0.1% of the estimated 2 million confirmed cases of Taiwan. Samples for RNA extraction were collected in 3 mL sterile viral transport medium (VTM) tubes and consisted of 2405 nasopharyngeal swabs belonging to 2339 patients. RNA was prepared by automated extraction using TANBead Nucleic Acid Extraction kits REF M665A46 (Taiwan Advanced Nanotech Inc.) and the QIAsymphony SP protocol (QIAGEN). This study was reviewed and approved by the Research Ethics Committee (110-110-E) of NTU-HCH.

COVIDseq

We carried out the sequencing using Illumina COVIDSeq Test kits (RUO version) according to the manufacturer's instructions. The workflow consists the following steps: cDNA synthesis, then virus target amplification using V3 nCov-2019 primers, followed by library preparation and library pooling. Subsequently, 98 SARS-CoV-2 targets and 11 human targets, the latter acting as controls, were analyzed on a NovaSeq 6000 instrument using 2x151-bp paired-end reads. Next, we used Illumina DRAGEN COVID Lineage app version 3.5.9 (base on pangolin 4.1.2 pangolin-data 1.12 and NextClade 1.11.0) in the BaseSpace Sequence Hub for rapid analysis.

Phylogenetic analysis

A set of 1966 NTU-HCH sequences were deposited to the Global Initiative on Sharing All Influenza Data (GISAID) (*1*) with the following epi accession numbers: EPI_ISL_14192849 to EPI_ISL_14192840, EPI_ISL_14191496 to EPI_ISL_14191488, EPI_ISL_14191320 to EPI_ISL_14190364, and EPI_ISL_14190353 to EPI_ISL_14189364. To investigate the global transmission, 228 BA.2.3.7, 277 global, and 376 additional sequences from Taiwanese were retrieved from GISAID as of July 2022. A total of 2847 sequences, including 1966 from NTU-HCH and 881 reference sequences (Appendix 2: GISAID Acknowledgment) that had met the quality standard, were used for the analysis. The phylogenetic tree was initially constructed using Nextstrain CLI (command-line interface) (version 3.2.5) (*2*) and annotated and visualized using ggtree package (*3*).

References

1. Shu Y, McCauley J. GISAID: Global initiative on sharing all influenza data - from vision to reality. Euro Surveill. 2017;22:30494. PubMed https://doi.org/10.2807/1560-7917.ES.2017.22.13.30494

2. Hadfield J, Megill C, Bell SM, Huddleston J, Potter B, Callender C, et al. Nextstrain: real-time tracking of pathogen evolution. Bioinformatics. 2018;34:4121–3. PubMed https://doi.org/10.1093/bioinformatics/bty407

3. Yu G, Smith DK, Zhu H, Guan Y, Lam TT-Y. ggtree: an r package for visualization and annotation of phylogenetic trees with their covariates and other associated data. Methods Ecol Evol. 2017;8:28–36. https://doi.org/10.1111/2041-210X.12628

Appendix 1 Table 1. Sequences selected for GISAID submission and phylogenetics analysis

		Coverage						
Batch	**Run**	**≥98**	**90-97**	**80-89**	**70-79**	**60-69**	**<60**	**Total**
1	1	77	12	0	4	0	1	94
2	2	63	0	5	3	4	18	93
3	3	43	14	4	2	5	38	106
4	4	140	17	2	3	0	30	192
5	5A	360	15	5	2	0	2	384
	5B	331	30	11	6	1	5	384
	5C	304	47	15	14	4	0	384
	5D	366	16	2	0	0	0	384
	5E	359	24	0	1	0	0	384
	NTU-HCH*	1966	163	44	31	14	93	2311
	Total†	2043	175	44	35	14	94	2405

*Count of NTU-HCH cases, from Batch (Run-2) through Batch 5 (Runs 5A-5E)
†Total count of all three hospitals

Appendix 1 Table 2. Samples and cases collected for this study as analyzed by the Illumina COVIDSeq system on NovaSeq6000

Batch	Sampling time period	Hospital source	Sample number	Case number	Sequencing run
1st	2021 (May)	WKH 12 CGMH 82	94	93	SP 2x51bp
2nd	2021 (May-July)	WKH 12* CGMH 82* NTU-HCH 93	93†	69†	S4 lane 2x151bp
3rd	2021 (Dec)~2022 (Feb) + 2020 (Nov)~2021 (April~Nov) 11 cases	NTU-HCH 106	106	66	S4 lane 2x151bp
4th	2022 (Feb 25~April 27) + April 27 (1 case)	NTU-HCH 192	192	191	S4 lane 2x151bp (288 sample/ lane)
5th	5A_2022 (April 26~May 3)	NTU-HCH 384	384	384	S4 lane 2x151bp
	5B_2022 (May 3~May 23)	NTU-HCH 384	384	384	S4 lane 2x151bp
	5C_2022 (May 17~June 6)	NTU-HCH 384	384	384	S4 lane 2x151bp
	5D_2022 (May 4~June 6)	NTU-HCH 384	384	384	S4 lane 2x151bp
	5E_2022 (May 18~June 6) + July 6 (1 case)	NTU-HCH 384	384	384	S4 lane 2x151bp

*Repeat from the first batch
†New samples from NTU-HCH

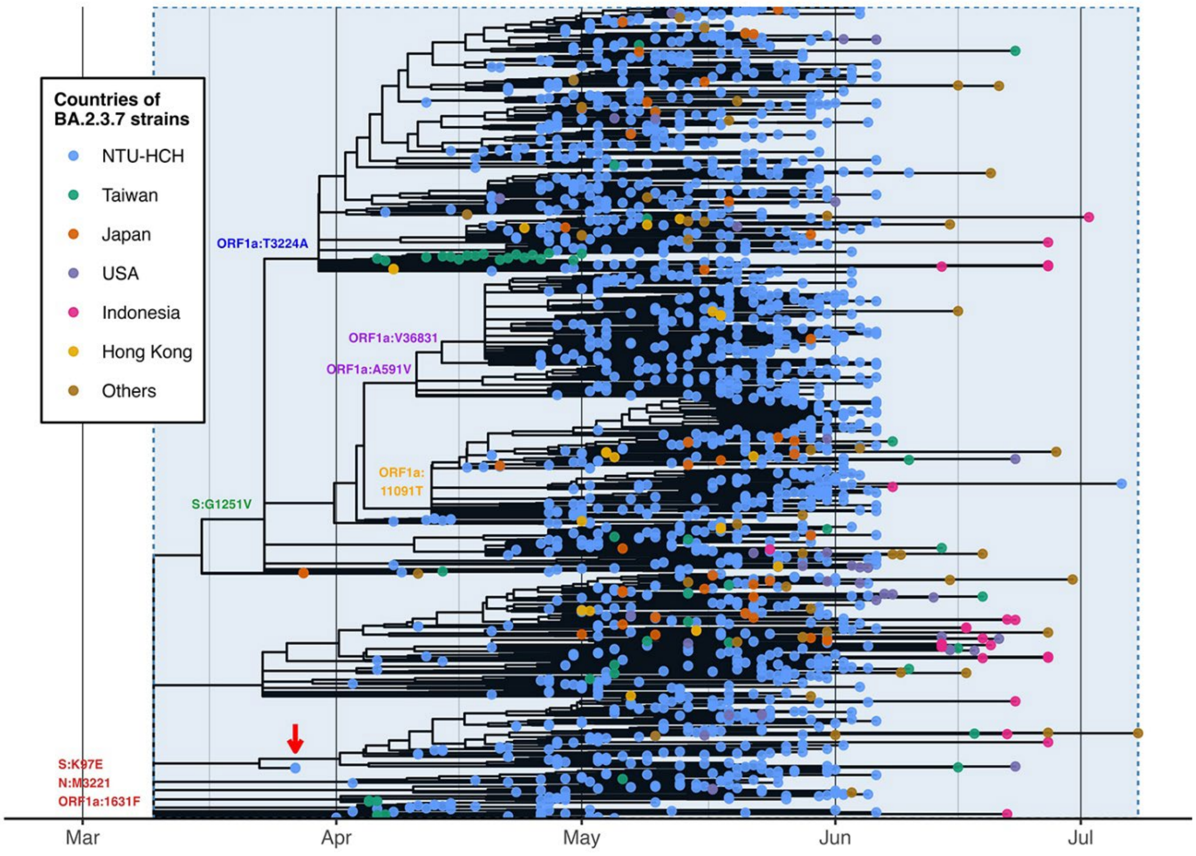

Appendix 1 Figure 1. Analysis of 1577 BA.2.3.7 sequences submitted to GISAID from the current study and 228 BA.2.3.7 sequences deposited to GISAID by other groups. The position of signature mutations are indicated: 3 (S:K97E, N:M322I, ORF 1a:L631F) are located at the origin of the B.A.2.3.7 lineages. S:G1251V is mapped at a major branch in the upper trunk of the tree, under which 3 minor branches can be defined by mutations in ORF1a:T3224A (in blue), ORF1a:A591V and ORF1a:V3683I (in purple), and ORF1a:I1091T (in orange). Sample origins are color coded. Red arrow denotes the index case collected from Taiwan on March 27, 2022.

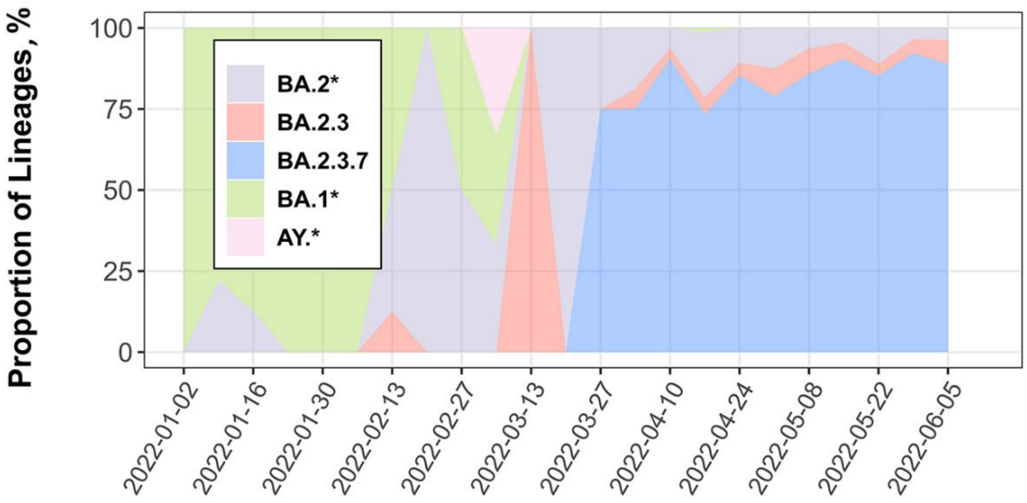

Appendix 1 Figure 2. Lineage distribution of National Taiwan University Hospital–Hsinchu Branch (NTU-HCH) strains. Most were identified as BA.1 or BA.2 lineages and sublineages annotated with an asterisk (*) before end of March. Since then, the BA.2.3.7 lineage became the dominant variant circulating in Taiwan.

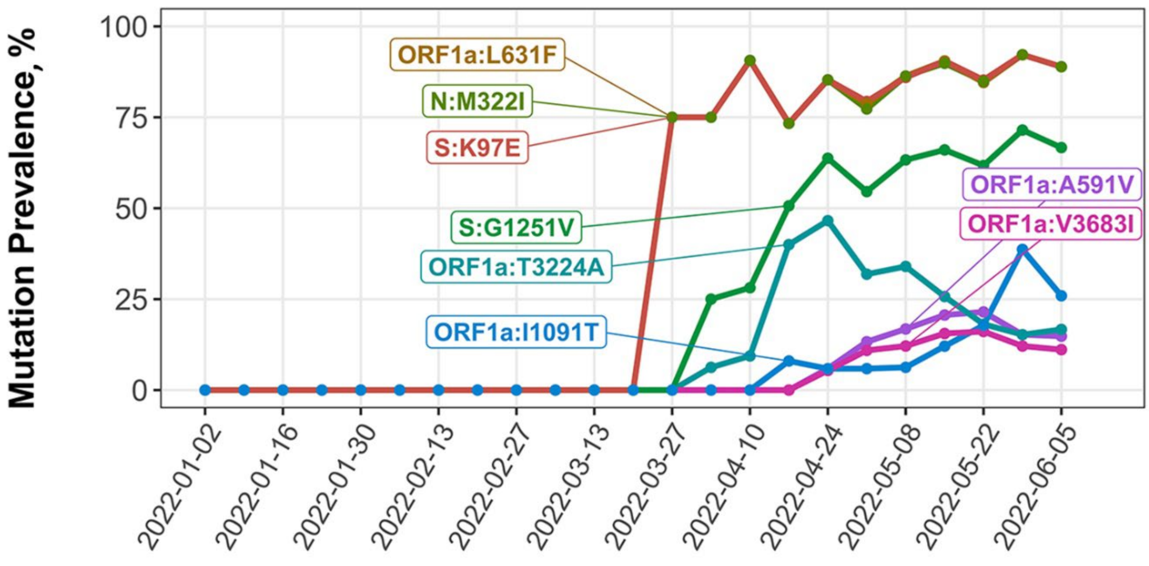

Appendix 1 Figure 3. Proportion of the different signature mutations in January 1–June 6, 2022 derived from a study of the Omicron BA.2.3.7 variant in Taiwan. Note the sharp increase of the Omicron variant 2.3.7 from week 13 (March 27– April 2) onward; the proportion of this Omicron variant remained high for at least 10 weeks. Also note overlapping of the 3 signature mutations (S:K97E, N:M322I, ORF1a:L631F) of BA.2.3.7 and a steady increase of sequences positive for S:G1251V from week 14 (April 3–9), reaching a plateau at week 17 (April 24–30).
